# Traumatic Cardiac Rupture at the Superior Vena Cava-Right Atrial Junction in Four Pediatric Patients: A Case Series in China National Children’s Medical Center

**DOI:** 10.1093/icvts/ivag032

**Published:** 2026-02-02

**Authors:** Jia Zheng, Zhiqiang Li, Song Bai, Jian Guo

**Affiliations:** Beijing Children’s Hospital, Capital Medical University, Beijing, China; Beijing Children’s Hospital, Capital Medical University, Beijing, China; Beijing Children’s Hospital, Capital Medical University, Beijing, China; Beijing Children’s Hospital, Capital Medical University, Beijing, China

**Keywords:** cardiac injury, pediatric trauma, echocardiography, cardiac rupture

## Abstract

This case series reports 4 paediatric patients (aged 2-11 years old) who sustained traumatic cardiac rupture following blunt trauma, all of whom were successfully treated at our institution. In contrast to common adult presentations, each case featured a rupture localized specifically to the superior vena cava-right atrial (SVC-RA) junction. Diagnosis was confirmed by echocardiography in the presence of cardiac tamponade. All patients underwent urgent median sternotomy, during which primary repair was accomplished without cardiopulmonary bypass. Postoperatively, both shock index and mean arterial pressure showed significant improvement (*P* = .003 and *P* = .028, respectively), and all children recovered fully without complications. This series highlights the SVC-RA junction as a uniquely vulnerable site in paediatric blunt cardiac injury and demonstrates that rapid diagnosis coupled with timely surgical intervention is paramount for survival.

## INTRODUCTION

Cardiac injury is associated with a high fatal condition. A timely diagnosis and appropriate treatment can substantially reduce mortality rates.^1^ Most reported cardiac trauma cases occur in adults due to motor vehicle accidents. Paediatric cases are rarer and may have different characteristics. Compared with published literature, our paediatric study demonstrated several unique features that merit reporting.

## CASE PRESENTATION

This investigation was approved by our hospital ethics committee. We conducted a retrospective study of 4 patients (aged 2-11 years, 3 females) treated between January 2007 and September 2019. Given that all cases involved emergency surgery and the study was retrospective in nature, the ethics committee waived the requirement for obtaining written informed consent. All patients sustained blunt cardiac injury from mechanisms including impact by heavy objects, falls, and a motorcycle accident. Upon transfer to our hospital (4-24 hours post-injury), all patients presented with clinical signs of cardiac tamponade. Echocardiography revealed a large pericardial effusion with clots and reduced inferior vena cava collapsibility. Haemodynamic parameters were unstable, with systolic blood pressure dropping in all cases.

All patients underwent urgent median sternotomy. Upon opening the pericardium, under pressure, a large volume of blood and clots was expelled. In all 4 cases, the rupture site was identified at the junction of the superior vena cava and the right atrium, approximately 10 mm in length. Bleeding was controlled by a right-angled vascular clamp to achieve partial occlusion at the SVC-RA junction, then Prolene suturing (**Video 1**). Cardiopulmonary bypass was not used. All patients recovered well and were discharged within 2 weeks with no delayed mortality or consciousness issues. Postoperative data showed significant improvement in shock index and mean arterial pressure (**Table 1**).

**Table 1.** Summary of Patient Characteristics and Clinical Parameters

Patient	Age/sex	Injury mechanism	Preop MBP/HR	Preop SI	Postop MBP/HR	Postop SI	Rupture site
1	2/F	Hit by metal stairs	54/190	1.64	68/132	0.66	SVC-RA
2	5/F	Hit by heavy gate	52/170	1.49	85/118	0.56	SVC-RA
3	9/F	Fall from height	65/170	1.02	75/108	0.58	SVC-RA
4	11/M	Motorcycle accident	59/180	1.00	84/121	0.52	SVC-RA

Abbreviations: HR, heart rate (bpm); MBP, mean blood pressure (mmHg); SI, shock index (HR/SBP); SVC-RA, superior vena cava-right atrium junction.

a Paired *t*-test results: Shock Index *P* = .003. Mean 1.34, 95% confidence interval 0.84 to 1.83 (statistically significant); Mean pressure *P* value = .028. Mean –20.34, 95% confidence interval –36.58 to –4.08 (statistically significant).

## DISCUSSION

The management of blunt cardiac injury in children presents considerable challenges, the foremost of which is the necessity for early and accurate diagnosis to guide life-saving intervention. Pericardial tamponade is a common manifestation of cardiac injury.^2^ It can be detected by X-ray computed tomography (CT) and echocardiography. Echocardiography is the most valuable tool for detecting pericardial effusion and tamponade.^3^ The progressively increasing pericardial effusion and the formation of blood clots can confirm the diagnosis of cardiac rupture. In our series, although it did not precisely identify the ruptured site.

In our practice, we discourage attempts to drain the blood into the mediastinal pleura. First, the pericardium is intact before drainage, and if the pressure is above the right atrium or the clot covers the bleeding hole, hypovolaemia could be controlled. In a child of approximately 5 years, the pleura cava can store nearly more than half of the total blood volume. Second, these patients are transferred to the hospital ER, which can take at least 4 hours and even up to 24 hours. These patients often maintain a precarious haemodynamic balance for hours after the initial injury, which can be disrupted by invasive procedures prior to definitive surgical control.

We emphasize that in cases with undefined associated injuries, systemic heparinization, then using cardiopulmonary bypass during repair carries the risk of bleeding in organs such as the brain, abdominal cavity, and pelvis.

In our cohort, the rupture was formed close to the right atrium and superior vena cava. This consistent rupture site represents a distinctive characteristic in our paediatric cohort and we found 2 plausible explanations. First, among the 4 cardiac chambers, the right atrium has the thinnest wall. This makes it more susceptible to rupture in response to increasing pressure. Blunt trauma exerts the strongest force on the chest wall and abdomen, leading to increased forces on the major veins in the chest and a significant elevation in the central venous pressure.^4^^,^^5^ This pressure is subsequently transmitted to the heart. Rupture of the right atrium would help alleviate the pressure and safeguard the other 3 chambers. Second, the heart is situated within the mediastinum and anchored by the major vessels. Blunt trauma can potentially displace the heart within the mediastinum, and the shear force can cause a tear in the heart. Compared with the aorta and left ventricle, the right atrium and superior vena cava are more fragile in nature. Shock index and mean pressure proved to be useful parameters, showing significant improvement post-surgery, reflecting successful resuscitation.

## CONCLUSION

Paediatric cardiac rupture from blunt trauma requires high clinical suspicion. Echocardiography is critical for early diagnosis. Emergency surgery via median sternotomy remains the definitive life-saving treatment. Cardiopulmonary bypass is just a backup treatment method. The SVC-RA junction is a vulnerable site in children. Prompt surgical intervention leads to excellent outcomes.

## Data Availability

The data that support our research are available from corresponding author upon request.
